# Primary cutaneous histoplasmosis difficult to treat in immunocompetent patient: case report and literature review

**DOI:** 10.31744/einstein_journal/2021RC5488

**Published:** 2021-05-13

**Authors:** Jéssica Mauricio Batista, Maria Auxiliadora Parreiras Martins, Caryne Margotto Bertollo

**Affiliations:** 1 Universidade Federal de Minas Gerais Faculdade de Farmácia Belo HorizonteMG Brazil Faculdade de Farmácia, Universidade Federal de Minas Gerais, Belo Horizonte, MG, Brazil.

**Keywords:** Histoplasmosis, Histoplasma, Immunocompetence, Itraconazole

## Abstract

Histoplasmosis is an infection caused by the dimorphic fungus *Histoplasma capsulatum*. The disease is endemic in several regions of tropical and temperate climate. The fungus presents opportunistic behavior, causing widespread infection in immunocompromised patients, resulting from complication of primary pulmonary infection, due to exogenous reinfection or reactivation of a quiescent source. In immunocompetent individuals, approximately 95% of pulmonary infections are asymptomatic. However, prolonged exposure to high amount spores may lead to acute or chronic lung infection. Due to the low amount of inoculum, primary cutaneous histoplasmosis caused by traumatic implantation is extremely rare and effectively treated with triazoles. Thus, the present study aims to report a case of primary cutaneous histoplasmosis that is difficult to treat in an immunocompetent patient, and to review the literature on the incidence of drug-resistant *Histoplasma capsulatum* strains in clinical practice.

## INTRODUCTION

The etiologic agent of histoplasmosis, *Histoplasma capsulatum*, is a dimorphic fungus found in the form of filamentous mycelium, with micro and macroconidia, in soil contaminated by bird and bat feces.^(^^1^^)^ The disease is endemic in tropical and temperate climate regions, such as in the river valleys of North and Central America, South America, eastern and southern Europe, eastern Asia, Africa, and Australia. In Brazil, the disease is present in all regions. The state of Rio de Janeiro, however, accounts for the largest number of microepidemics. Outbreaks of histoplasmosis have been associated with activities that disturb contaminated soil, favoring the inhalation of spores.^(^^2^^,^^3^^)^

In immunocompromised patients, histoplasmosis often presents as disseminated disease resulting from a complication of a primary pulmonary infection, due to exogenous reinfection or reactivation of a quiescent source. In these patients, skin lesions occur in 4% to 11% of cases and result from secondary invasion of the skin due to hematogenic dissemination of infected macrophages.^(^^1^^)^ The mortality associated with untreated disseminated histoplasmosis is 80%. However, it can be reduced to 2% with appropriate therapy. For moderately severe to severe cases, liposomal amphotericin B (3mg/kg/day) is recommended for 1 to 2 weeks, followed by itraconazole (200mg orally) three times daily, for 3 days, and then twice daily, for at least 12 months (level of evidence AI). For mild to moderate cases, the use of amphotericin B is not necessary (level of evidence AII).^(^^3^^)^

In contrast, in immunocompetent individuals, approximately 95% of pulmonary infections are asymptomatic.^(^^1^^)^ Nevertheless, prolonged exposure to a high amount of spores can lead to an acute but self-limited pulmonary infection. In individuals over 50 years, who smoke, diagnosed with chronic obstructive pulmonary disease (COPD), the lung infection can slowly progress to the chronic fibrocavitary form.^(^^2^^)^ In such cases, the use of itraconazole with the same dosage as mentioned above is indispensable, achieving an effective response in 80% of cases (level of evidence AII). However, the duration of treatment can increase from 12 to 24 months because of the risk of relapse.^(^^3^^)^

Due to the low amount of inoculum, the occurrence of primary cutaneous histoplasmosis (PCH) by traumatic implantation, is extremely rare in non-immunocompromised patients and is effectively treated with triazoles.^(^^1^^)^ Thus, the aim of the present study is to report a case of difficult-to-treat PCH case in an immunocompetent patient with no history of previous exposure to risky environments, such as caves and poultry facilities, and to review the literature on the incidence of *H. capsulatum* strains resistant to drugs used in clinical practice.

## CASE REPORT

**Figure 1:**
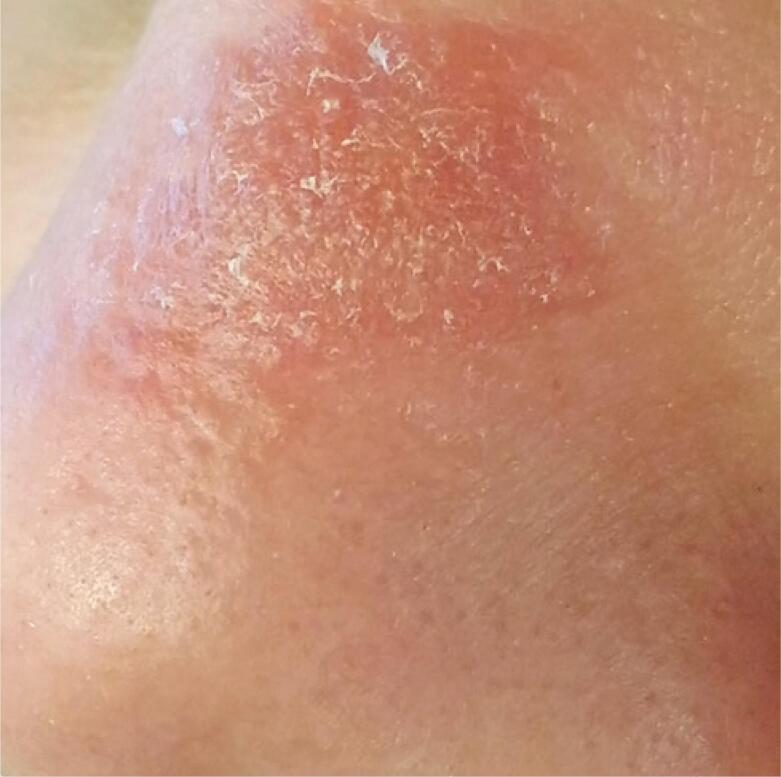
Erythematous papular lesion

A 26-year-old female patient, brown-skin, born, and living in Belo Horizonte (MG), presented with an erythematous papular lesion with irregular borders and centrifugal growth, on the dorsal region of the nose, measuring approximately 1cm in diameter (Figure 1). The skin lesion developed after the removal of a comedone, without the use of aseptic techniques, 4 months before clinical treatment. There was no history of systemic symptoms, significant past diseases, or use of relevant medicines. Serological tests (anti-hepatitis C, anti-hepatitis B, VDRL, and anti-HIV) were negative. Blood tests (complete blood count, lipid profile, prothrombin time and activity, activated partial thromboplastin time, and fasting glucose) showed no abnormalities.

**Figure 2:**
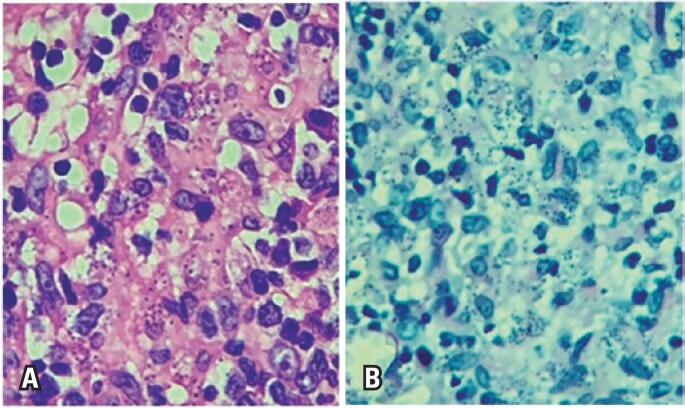
*Histoplasma capsulatum* yeast form stained by periodic acid Schiff (A) and Grocott-Gomori methenamine silver (B)

The macroscopic analysis of the incisional biopsy of the lesion showed a skin fragment measuring 0.6x0.4x0.3cm, with an irregular epidermal surface. Histopathological examination revealed a chronic, diffuse inflammatory process, with dense lympho-plasma-histiocytic infiltrate (Figure 2). A periodic acid Schiff stain (PAS) and a Grocott-Gomori's methenamine silver stain showed numerous spore-like fungal structures stained in red and dark brown, respectively. The fungi showed remarkably regular morphology, presenting as small, well-defined, slightly oval yeasts, approximately 2mm to 4mm in size, forming rows.

**Figure 3:**
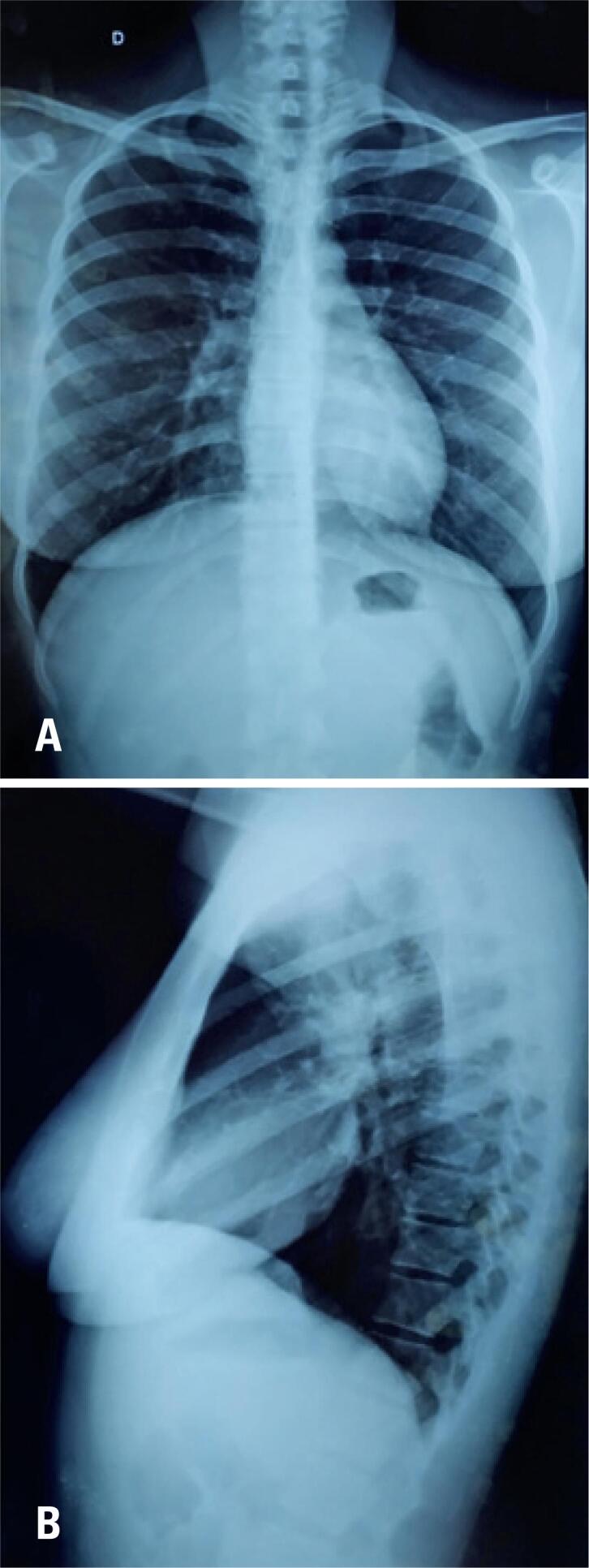
Plain chest radiography in posteroanterior (A) and lateral (B) views

Conventional chest radiography revealed clear lungs with no signs of pleuropulmonary injury, well-configured hila, free costophrenic angles, normal cardiothoracic index, and unaltered bone structure (Figure 3). No characteristic lesions of acute pulmonary histoplasmosis were found, such as presence of reticulonodular infiltrates accompanied by hilar and paratracheal lymphadenopathy.

The diagnosis of PCH was based on the history of traumatic inoculation with subsequent development of a local lesion, identification of the etiologic agent by histopathological examination, and absence of clinical and laboratory evidence of prior systemic or pulmonary infection. Treatment with oral itraconazole, 400mg a day, was initiated. Complete remission of the lesion occurred after 14 months of uninterrupted treatment. There were no significant changes in the serum indicators of liver damage during treatment - aspartate aminotransferase (AST), alanine aminotransferase (ALT), gamma glutamyl transferase (GGT), and alkaline phosphatase enzymes.

The study was approved by the Research Ethics Committee of *Universidade Federal de Minas Gerais* (CAAE: 37956820.2.0000.5149, parecer 4.383.903) and the Informed Consent Terms were signed by those responsible.

## DISCUSSION

The clinical manifestations of PCH can be similar to other infectious diseases. Therefore, the diagnosis depends mainly on the identification of the etiologic agent in histopathological examinations and/or culture, in addition to clinical and laboratory proof of the absence of prior pulmonary or systemic infection.^(^^1^^)^ It is worth mentioning that some authors have proposed the use of the Wilson criteria to distinguish PCH from secondary cutaneous histoplasmosis.^(^^4^^,^^5^^)^ However, over time, some caveats have been raised: the histoplasmin skin test does not distinguish between current and previous infections; not all lesions present with a chancre form appearance and are accompanied by lymphadenopathy; and, in immunocompromised patients, serum titers decrease or disappear with disease progression.^(^^1^^)^

Indeed, in individuals with an intact immune system, PCH is a rare clinical condition, mainly due to non-progression of infection.^(^^6^^)^ Saheki et al.^(^^1^^)^ performed an extensive literature review, finding only eight cases since the first, described by Curtis and Cawley, in 1947. Between 2008 and 2018, five new cases of the disease were reported,^(^^1^^,^^6^^–^^9^^)^ and none was associated with immunosuppression. Of the 13 cases reported in the literature, six showed spontaneous remission,^(^^1^^)^ two were mistreated with antibiotics,^(^^1^^)^ two were treated with liposomal amphotericin B,^(^^7^^,^^9^^)^ and three with itraconazole.^(^^1^^,^^6^^,^^9^^)^

Among the cases treated with antifungal drugs (Table 1), complete remission of the lesions occurred within a maximum of 8 weeks of treatment. The ability to eradicate infections in immunocompetent patients, the progression of the lesion, and the unusual need for prolonged treatment (14 months of uninterrupted treatment with itraconazole) make the present case report noteworthy.

**Table 1:** Studies reporting cases of primary cutaneous histoplasmosis in immunocompetent patients treated with liposomal amphotericin B and/or triazoles

Study	Type	Sex	Age (years)	Treatment	Lesion	Remission (weeks)	Comments
Saheki et al.^(^^1^^)^	Case report and review	Male	45	Itraconazole	Single	6	Patient with erythematous papular-tuberous lesion on the dorsum of the right hand. Treatment with itraconazole (400mg/day, PO)
Paixão et al.^(^^6^^)^	Case report	Male	70	Itraconazole	Multiple	4	Patient with erythematous genital lesions. Treatment with itraconazole (200mg/day, PO)
Raina et al.^(^^7^^)^	Case report	Male	32	Liposomal amphotericin B	Single	3	Patient with ulcerated lesion on the right thigh. Treatment with liposomal amphotericin B (3mg/kg/day)
Bhattacharya et al.^(^^8^^)^	Case report	Male	70	Fluconazole	Multiple	NR	Patient with nodular lesions on the face, head, neck, and trunk. Posology not reported.
Patra et al.^(^^9^^)^	Case report	Male	60	Liposomal amphotericin B and itraconazole	Multiple	8	Patient with erythematous papular lesions on the trunk, face, and upper limbs. Treatment with liposomal amphotericin B (3mg/kg/day) for 8 days, followed by itraconazole (400mg/day, PO)

NR: not reported; PO: *per os*.
