# Supplementary material for: Primary cutaneous histoplasmosis difficult to treat in immunocompetent patient: case report and literature review
Source: Einstein (Sao Paulo). 2021 May 13;19:eRC5488. doi: 10.31744/einstein_journal/2021RC5488 (PMC8121373; doi:10.31744/einstein_journal/2021RC5488)
Supplement: Translation

# Translation: Histoplasmose cutânea primária de difícil tratamento em paciente imunocompetente: relato de caso e revisão da literatura

**Authors:** Jéssica Mauricio Batista, Maria Auxiliadora Parreiras Martins, Caryne Margotto Bertollo

**Affiliations:** 1 Belo HorizonteMG Brasil Faculdade de Farmácia, Universidade Federal de Minas Gerais, Belo Horizonte, MG, Brasil.

**Keywords:** Histoplasmose, Histoplasma, Imunocompetência, Itraconazol

## Abstract

A histoplasmose é uma infecção causada pelo fungo dimórfico *Histoplasma capsulatum*. A doença é endêmica em diversas regiões de clima tropical e temperado. O fungo apresenta comportamento oportunístico, causando infecção disseminada em pacientes imunocomprometidos, resultante da complicação da infecção pulmonar primária, por reinfecção exógena ou reativação de um foco quiescente. Em indivíduos imunocompetentes, cerca de 95% das infecções pulmonares são assintomáticas. No entanto, a exposição prolongada à quantidade elevada de esporos pode levar à infecção pulmonar aguda ou crônica. Devido à baixa quantidade de inóculo, a histoplasmose cutânea primária causada por implantação traumática é extremamente rara e efetivamente tratada com triazóis. Assim, o presente estudo tem como objetivos relatar um caso de histoplasmose cutânea primária de difícil tratamento em paciente imunocompetente, e revisar a literatura a respeito da incidência de cepas de *Histoplasma capsulatum* resistentes aos fármacos utilizados na prática clínica.

## INTRODUÇÃO

O agente etiológico da histoplasmose, *Histoplasma capsulatum*, é um fungo dimórfico, encontrado em solo contaminado por fezes de aves e morcegos, na forma de micélio filamentoso com micro e macroconídios.^(^^1^^)^ A doença é endêmica em regiões de clima tropical e temperado, como nos vales fluviais das Américas do Norte e Central, na América do Sul, no leste e sul da Europa, no leste da Ásia, na África e na Austrália. No Brasil, a doença incide em todas as regiões, entretanto, o estado do Rio de Janeiro é responsável pelo maior número de microepidemias. Os surtos de histoplasmose têm sido associados a atividades que perturbam o solo contaminado, o que favorece a inalação de esporos.^(^^2^^,^^3^^)^

Em pacientes imunocomprometidos, a histoplasmose apresenta-se, frequentemente, sob a forma disseminada, resultado da complicação da infecção pulmonar primária, decorrente da reinfecção exógena ou da reativação de um foco quiescente. Nesses pacientes, as lesões cutâneas ocorrem em 4% a 11% dos casos e resultam da invasão secundária da pele devido à disseminação hematogênica de macrófagos parasitados.^(^^1^^)^ A mortalidade sem tratamento é de 80%. Porém, pode ser reduzida para 2% com terapia adequada. Para os casos moderadamente graves a graves, recomenda-se o uso de anfotericina B lipossomal (3mg/kg/dia) por 1 a 2 semanas, seguido de itraconazol (200mg, por via oral), três vezes ao dia, por 3 dias e, posteriormente, duas vezes ao dia por, no mínimo, 12 meses (nível de evidência AI). Para casos leves a moderados, o uso de anfotericina B é prescindível (nível de evidência AII).^(^^3^^)^

Em contrapartida, em indivíduos imunocompetentes, cerca de 95% das infecções pulmonares são assintomáticas.^(^^1^^)^ Todavia, a exposição prolongada a uma quantidade elevada de esporos pode levar à infecção pulmonar aguda, porém autolimitada. Em indivíduos tabagistas, com mais de 50 anos de idade e diagnosticados com doença pulmonar obstrutiva crônica (DPOC), a infecção pulmonar pode progredir lentamente para a forma fibrocavitária crônica.^(^^2^^)^ Nesses casos, o uso de itraconazol com posologia idêntica a citada é indispensável, obtendo resposta efetiva em 80% dos casos (nível de evidência AII). Contudo, o tempo de tratamento pode aumentar de 12 para 24 meses, por causa do risco de recaída.^(^^3^^)^

Devido à baixa quantidade de inóculo, a ocorrência de histoplasmose cutânea primária (HCP) por implantação traumática, em pacientes imunocompetentes, é extremamente rara e efetivamente tratada com triazóis.^(^^1^^)^ Assim, o presente estudo tem como objetivos relatar um caso de HCP de difícil tratamento em paciente imunocompetente, sem histórico de exposição prévia a ambientes de risco, como grutas e instalações avícolas, e revisar a literatura a respeito da incidência de cepas de *H. capsulatum* resistentes aos fármacos utilizados na prática clínica.

## RELATO DE CASO

Paciente do sexo feminino, 26 anos, parda, natural e residente em Belo Horizonte (MG). Apresenta lesão eritêmato-papulosa, com bordas irregulares e crescimento centrífugo, na região dorsal do nariz, medindo aproximadamente 1cm de diâmetro (Figura 1). A lesão cutânea surgiu após a remoção de um comedão, sem o emprego de técnicas assépticas, 4 meses antes do atendimento clínico. Não havia história de sintomas sistêmicos, doenças prévias significativas ou uso de medicamentos relevantes. Os exames sorológicos (anti-hepatite C, anti-hepatite B, VDRL e anti-HIV) foram negativos. Os exames de sangue (hemograma completo, lipidograma, tempo e atividade de protrombina, tempo de tromboplastina parcial ativado e glicemia de jejum) não apresentaram alterações.

**Figura 1:** Lesão eritêmato-papulosa

A análise macroscópica de biópsia incisional da lesão evidenciou fragmento de pele medindo 0,6x0,4x0,3cm, com superfície epidérmica irregular. O exame histopatológico revelou processo inflamatório crônico e difuso, com denso infiltrado linfo-plasmo-histiocitário (Figura 2). Foram observadas numerosas estruturas fúngicas com esporulação, coradas pelo ácido periódico de Schiff (PAS), apresentando-se na cor vermelha, e pela metenamina de prata de Gomori-Grocott, apresentando-se na cor marrom-escura. Os fungos exibiram morfologia com notável regularidade, apresentando-se sob forma de leveduras pequenas, levemente ovaladas, com aproximadamente 2mm a 4mm de tamanho, formando fileiras, bem definidas.

**Figura 2:** Forma leveduriforme de *Histoplasma capsulatum* corada pelo ácido periódico de Schiff (A) e pela prata metenamina de Gomori-Grocott (B)

A radiografia convencional do tórax revelou pulmões transparentes, sem sinais de lesão pleuropulmonar, hilos bem configurados, seios costofrênicos livres, índice cardiotorácico normal e estrutura óssea sem alterações (Figura 3). Não foram encontradas lesões características de histoplasmose pulmonar aguda, como presença de infiltrados reticulonodulares acompanhados de linfadenopatia hilar e paratraqueal.

**Figura 3:** Radiografia simples do tórax em posição posteroanterior (A) e lateral (B)

O diagnóstico de HCP foi embasado na história de inoculação traumática, com o desenvolvimento subsequente de lesão local, identificação do agente etiológico por meio de exame histopatológico e ausência de evidências clínicas e laboratoriais de infecção sistêmica ou pulmonar prévia. Foi iniciado o tratamento com itraconazol, 400mg ao dia, via oral. A remissão completa da lesão ocorreu após 14 meses de tratamento ininterrupto. Não houve alterações significativas nos indicadores séricos de lesão hepática durante o tratamento – enzimas aspartato aminotransferase (AST), alanina aminotransferase (ALT), gamaglutamiltransferase (GGT) e fosfatase alcalina.

O estudo foi aprovado pelo Comitê de Ética em Pesquisa da Universidade Federal de Minas Gerais (CAAE: 37956820.2.0000.5149, parecer 4.383.903) e o Termo de Consentimento Livre Esclarecido foi assinado pela paciente.

## DISCUSSÃO

As manifestações clínicas da HCP podem ser confundidas com diversas doenças infecciosas. Dessa forma, o diagnóstico depende, principalmente, da identificação do agente etiológico em exames histopatológicos e/ou meio de cultura, além da comprovação clínica e laboratorial da ausência de infecção pulmonar ou sistêmica prévia.^(^^1^^)^ É importante ressaltar que alguns autores propuseram a utilização dos critérios de Wilson para distinguir a HCP da histoplasmose cutânea secundária.^(^^4^^,^^5^^)^ Todavia, com o passar do tempo, algumas ressalvas foram feitas: o teste cutâneo da histoplasmina não distingue a infecção atual das precedentes; nem todas as lesões apresentam aspecto cancriforme e são acompanhadas de linfadenopatia e, em pacientes imunocomprometidos, os títulos sorológicos decrescem ou desaparecem com a progressão da doença.^(^^1^^)^

De fato, a HCP, em indivíduos com sistema imunológico intacto, é uma entidade clínica rara, principalmente devido a não progressão da infecção.^(^^6^^)^ Saheki et al.^(^^1^^)^ realizaram extensa revisão da literatura, encontrando apenas oito casos, desde o primeiro, descrito por Curtis e Cawley, em 1947. Entre 2008 e 2018, foram relatados cinco novos casos da doença,^(^^1^^,^^6^^–^^9^^)^ e nenhum estava associado à imunossupressão. Dos 13 casos relatados na literatura, seis apresentaram remissão espontânea,^(^^1^^)^ dois foram tratados erroneamente com antibióticos,^(^^1^^)^ dois foram tratados com anfotericina B lipossomal^(^^7^^,^^9^^)^ e três com itraconazol.^(^^1^^,^^6^^,^^9^^)^

Entre os casos tratados com antifúngicos (Tabela 1), a remissão completa das lesões ocorreu, em, no máximo, 8 semanas de tratamento. Devido à capacidade de erradicação das infecções em pacientes imunocompetentes, a progressão da lesão e a necessidade incomum de tratamento prolongado (14 meses de tratamento ininterrupto com itraconazol) tornam o presente relato de caso digno de nota.

**Tabela 1:** Estudos que relataram casos de histoplasmose cutânea primária em pacientes imunocompetentes tratados com anfotericina B lipossomal e/ou triazóis

Estudo	Tipo	Sexo	Idade (anos)	Tratamento	Lesão	Remissão (semanas)	Comentários
Saheki et al.^(^^1^^)^	Relato de caso e revisão	Masculino	45	Itraconazol	Única	6	Paciente com lesão eritêmato-pápulo-tuberosa no dorso da mão direita. Tratamento com itraconazol (400mg/dia, VO)
Paixão et al.^(^^6^^)^	Relato de caso	Masculino	70	Itraconazol	Múltiplas	4	Paciente com lesões genitais eritematosas. Tratamento com itraconazol (200mg/dia, VO)
Raina et al.^(^^7^^)^	Relato de caso	Masculino	32	Anfotericina B lipossomal	Única	3	Paciente com lesão ulcerada na coxa direita. Tratamento com anfotericina B lipossomal (3mg/kg/dia)
Bhattacharya et al.^(^^8^^)^	Relato de caso	Masculino	70	Fluconazol	Múltiplas	NR	Paciente com lesões nodulares na face, cabeça, pescoço e tronco. Posologia não relatada.
Patra et al.^(^^9^^)^	Relato de caso	Masculino	60	Anfotericina B lipossomal e itraconazol	Múltiplas	8	Paciente com lesões pápulo-eritematosas no tronco, face e membros superiores. Tratamento com anfotericina B lipossomal (3mg/kg/dia) durante 8 dias, seguido de itraconazol (400mg/dia, VO)

NR: não relatado; VO: via oral.
